# Supplementary material for: Direct observation of the complex S(IV) equilibria at the liquid-vapor interface
Source: Nat Commun. 2024 Oct 18;15:8987. doi: 10.1038/s41467-024-53186-5 (PMC11487263; doi:10.1038/s41467-024-53186-5)
Supplement: Supplementary file 1 — Supplementary Information [file 41467_2024_53186_MOESM1_ESM.pdf]

Supplementary Information to: "Direct observation of the complex S(IV) equilibria at the liquid-vapor interface"

Tillmann Buttersack[1*†], Ivan Gladich[2*†], Shirin Gholami[1], Clemens Richter[1], Rémi Dupuy[3], Christophe Nicolas[4], Florian Trinter[1], Annette Trunschke[1], Daniel Delgado[1], Pablo Corral Arroyo[5], Evelyne A. Parmentier[5], Bernd Winter[1], Lucia Iezzi[6], Antoine Roose[6, 7], Anthony Boucly[6], Luca Artiglia[6], Markus Ammann[6], Ruth Signorell[5], Hendrik Bluhm[1*]

[1*]Fritz Haber Institute of the Max Planck Society, Faradayweg 4–6, D-14195 Berlin, Germany.
[2*]Qatar Environment and Energy Research Institute, Hamad Bin Khalifa University, P.O. Box 31110, Doha, Qatar.
[3]Sorbonne Université, CNRS, Laboratoire de Chimie Physique—Matière et Rayonnement, Paris Cedex 05, F-75005, France.
[4]Synchrotron SOLEIL, L'Orme des Merisiers, Gif-sur-Yvette, Saint-Aubin—BP 48 91192, France.
[5]Laboratory of Physical Chemistry, Department of Chemistry and Applied Biosciences, ETH Zürich, Vladimir-Prelog-Weg 2, Zürich, CH-8093 , Switzerland.
[6]PSI Center for Energy and Environmental Sciences, Paul Scherrer Institute, Villigen PSI, CH-5232, Switzerland.
[7]Current address: IMT Nord Europe, Institut Mines-Télécom, University Lille, F-59000, France.

*Corresponding author(s). E-mail(s): buttersack@fhi.mpg.de; igladich@hbku.edu.qa; bluhm@fhi.mpg.de;
†These authors contributed equally to this work.

1 X-ray photoelectron spectroscopy

1.1 Reference measurements

In addition to the measurements of sulfite solutions, we performed experiments with sulfate and methyl sulfonate (see also Tab. 1 of main the manuscript). Figure 1 shows the S 2p spectrum of a sodium sulfate solution (400 mM) and a mixed solution of sodium sulfate and sulfite (200 + 200 mM) compared to a sulfite solution.

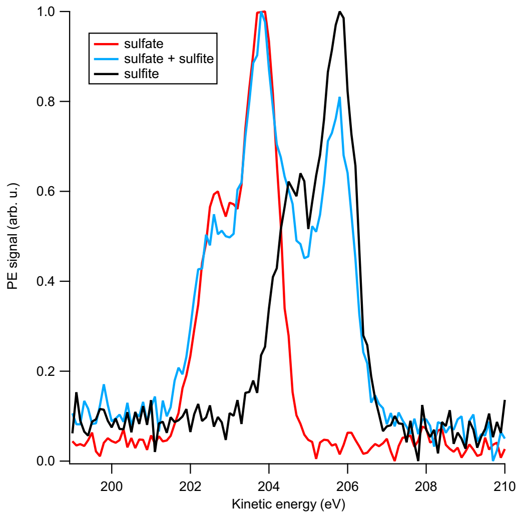

Supplementary Figure 1 Normalized photoelectron spectra of sodium sulfate, sodium sulfite, and a 1:1 mixture thereof (total concentration in all cases 400 mM), measured at a photon energy of 377 eV.

Figure 2 shows the S 2p spectrum of a sodium sulfate (400 mM, pH=7) and a sodium methyl sulfonate solution. The peak attributed to sulfonate in Fig. 2 of the main manuscript has a similar BE than the one found for methyl sulfonate.

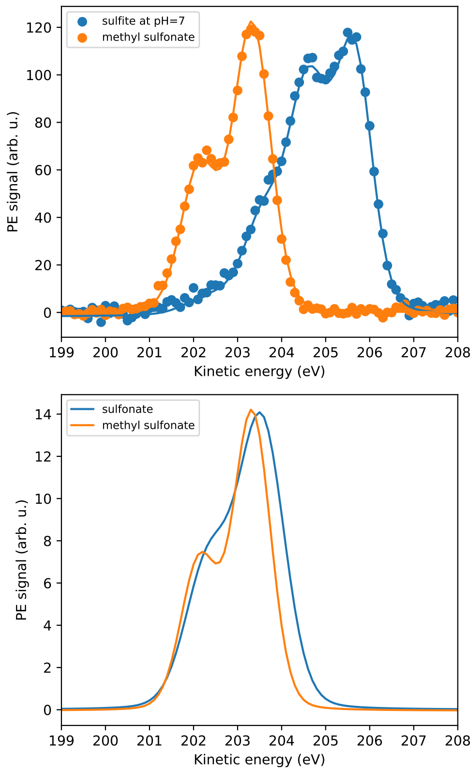

Supplementary Figure 2 Top panel: S 2p spectra of a sodium sulfite solution (pH=7) and a methyl sulfonate solution. Bottom panel: The fit of the methane sulfonate (orange) is very similar to the peak attributed to sulfonate (blue). The peaks of bisulfite and sulfite are depicted in Fig. 2 of the main manuscript.

1.2 Estimation of the concentration of dissolved sulfur dioxide

In Fig. 4 in the main manuscript we used Henry's law (Eq. 1) and the measured peak area ratio of the gases $SO_{2(g)}$ and $H_2O_{(g)}$ to estimate the fraction of dissolved $SO_{2(aq.)}$.[1]

$$H_i^{cp} = \frac{c_{i,aq.}}{p_i} \tag{1}$$

At 283 K (the injection temperature of the liquid jet) the Henry's law constants are $H_{H_2O} = 45.5$ mol m^{-3} Pa^{-1} and $H_{SO_2} = 0.122$ mol m^{-3} Pa^{-1}[2, 3], while at 273 K $H_{H_2O} = 91$ mol m^{-3} Pa^{-1} and $H_{SO_2} = 0.116$ mol m^{-3} Pa^{-1}. The exact temperature of the solution at the point of measurement is unknown. However, temperature measurements on flatjets revealed that the temperature drops about 5 K within the first mm downstream after injection.[4] Therefore, we used a temperature of 5 degree Celsius (± 5 K) for the ratio of the Henry's law constants:

$$H_{ratio} = \frac{H_{H_2O}}{H_{SO_2}} = 580 \pm 200. \tag{2}$$

We assume the same pressure gradients for SO_2 and H_2O in the gas phase surrounding the jet. Therefore,

$$\frac{p_{H_2O}}{p_{SO_2}} = \frac{A_{H_2O_{(g)}}}{A_{SO_{2(g)}}} \tag{3}$$

where A is the area of the gas phase peaks. In combination with Eq. 1, we get

$$H_{ratio} = \frac{A_{SO_{2,(g)}}}{A_{H_2O_{(g)}}} \cdot \frac{c_{H_2O_{(aq.)}}}{c_{SO_{2,(aq.)}}}. \tag{4}$$

Rearranging Eq. 4 leads to:

$$c_{SO_{2(aq.)}} = \frac{c_{H_2O}}{H_{ratio}} \cdot \frac{A_{SO_{2(g)}}}{A_{H_2O_{(g)}}}. \tag{5}$$

1.2.1 Detection of dissolved sulfur dioxide

The signal-to-noise (S/N) in the spectrum at pH 0.36 where $SO_{2,(aq.)}$ was detected by applying a negative potential to the solution was rather poor. To prove that there is indeed an additional peak due to $SO_{2,(aq.)}$ we have fitted the data depicted in the manuscript again with only one component, the sulfonate peak. The disagreement of fit and data is indicating that a further species is contributing to the overall signal. This component is attributed to $SO_{2,(aq.)}$, in agreement with the theoretical calculations of the BE shifts (Table 1 in the main manuscript).

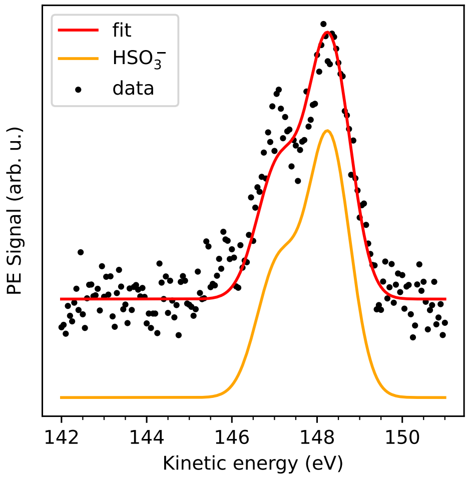

Supplementary Figure 3 Same data as depicted in Fig. 2f in the main manuscript, but fitted with only one component.

1.2.2 Calculation of absolute concentrations

To avoid saturation of the electron detector when recording the O 1s reference spectra, we reduced the photon flux to about 1% by inserting baffles at beamline P04, PETRA III, DESY. The S 2p spectra were recorded with the identical baffle settings to be able to compare the intensities of the spectra directly. Additionally, we recorded the S 2p spectra with the baffle settings for the maximum photon flux to have optimum S/N conditions; these spectra are depicted in the main manuscript (Fig. 2). The reduction of the intensity of the S 2p spectra due to the baffles was calculated by comparing the total signals from the two settings, leading to the factor $f_{baffles}$.

The intensity of the XPS signal is proportional to the photon flux F, the photoemission cross section σ, and anisotropy parameter β of the O 1s and S 2p features. The flux was measured with a photodiode (SXUV100). The spectral responsivity of the photodiodide is a function of the photon energy (0.24 A/W for 738 eV and 0.17 A/W for 377 eV).[5] The cross sections were taken from Yeh and Lindau.[6] The measurements were performed at angles close to the magic angle, such that the anisotropy factor can be neglected here. We used the following equation to calculate the concentration c_i of the species i:

$$c_i = c_{water} \cdot \frac{A_i}{A_{water}} \cdot \frac{1}{f_{baffles}} \cdot \frac{F_{O1s} \cdot \sigma_{O1s}}{F_{S2p} \cdot \sigma_{S2p}} \tag{6}$$

where A_i is the corresponding peak area. The total concentration of sulfur species was calculated with an error of about 10% (see Fig. 4d in the main manuscript) basing on the sweep to sweep stability.

Data recorded at SOLEIL did not require to reduce the photon flux for O 1s spectra. There, an AXUV100G photodiode was used. However, there was some uncertainty

about the spectral responsivity of the photodiode with time.[5] For the SOLEIL data we used the O 1s spectra of a sulfite solution with a pH of 12 to get an intrinsic calibration, as in this case the O 1s signal of SO_3^{2-} is clearly separated from the O 1s water signal. An example spectrum is shown in Fig. 4. The binding energy of O 1s of sulfite is 2.25 eV lower than that of liquid water. Using the known O 1s binding energy of water as a reference, we report the binding energy of the O 1s of sulfite as 535.85 ± 0.05 eV.[7]

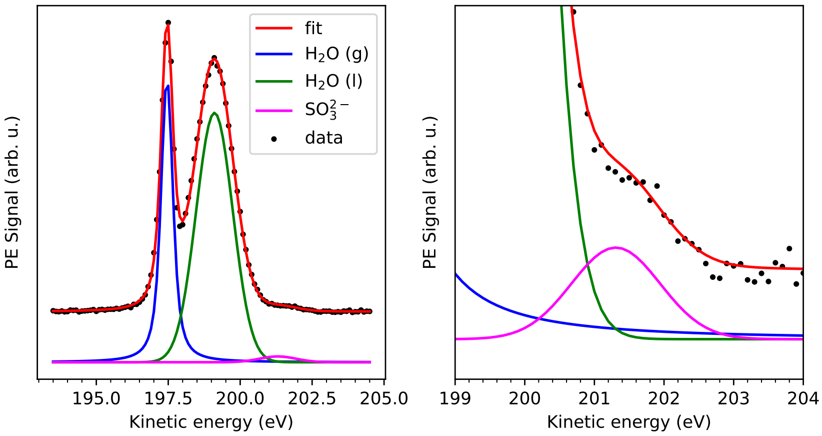

Supplementary Figure 4 O 1s spectrum of a 400 mM sulfite solution (pH=12). The right panel is an enlarged version of the left one. The signal of sulfite (pink) is separated from the gas phase and liquid phase water signals (blue and green lines, respectively).

The concentration of sulfite can then directly be calculated from the O 1s peak areas, as flux, cross section and beta parameters are canceling out. However, one has to consider that one sulfite molecule contains three oxygen atoms:

$$c_{SO_3^{2-}} = c_{water} \cdot 1/3 \cdot \frac{A_{SO_3^{2-}}}{A_{H_2O}} = 450 \pm 80 \text{ mM} \tag{7}$$

The error bar was determined from the standard deviation of 7 spectra and is about of 7%. Additionally, one has to consider that the O 1s peak of liquid water is about 100 times larger and partially overlaps with the sulfite signal. Increasing the width of the water-peak by 0.025 eV leads to a 10% decrease of the sulfite peak. Taking this into account we arrive at an error bar of 17%.

1.3 Valence-band XPS spectra

In addition to the core-level spectra shown and discussed in the main manuscript, we measured the valence-band spectra for sulfite solutions (400 mM) at three pHs (4, 7,

and 10), which are depicted in Fig. 5. Sulfite can also be detected in the valence band, and the spectral features at around 7-9 eV BE clearly depend on the pH. However, unlike in the analysis of S 2p spectra, the spectral features cannot easily attributed to certain species. Nevertheless, there are some trends. First, the peak around 9.3 eV (red) is decreasing with increasing pH. Second, the peak around 7.5 eV (orange) is absent in the case of pH 4 where only protonated species are present.

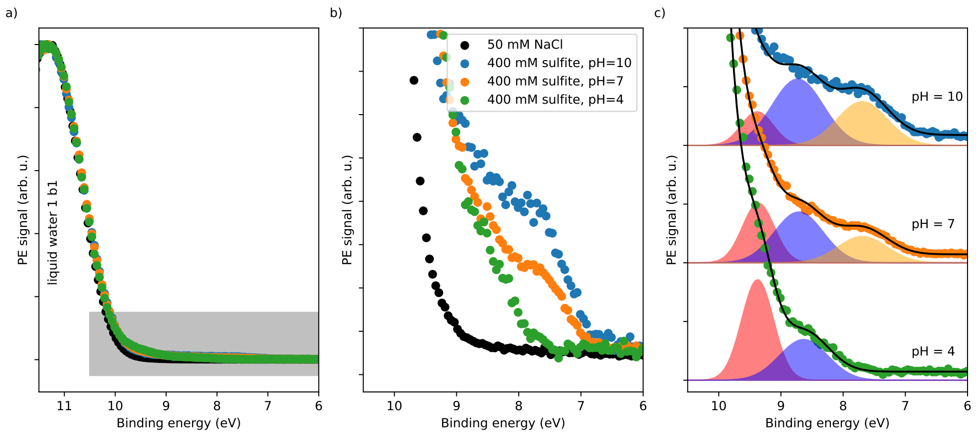

Supplementary Figure 5 Valence-band spectra of sulfite solutions (400 mM) at three different pHs recorded with a photon energy of 210 eV. For comparison the valence band spectrum of a 50 mM NaCl solution is plotted as well (black). **a)** VB spectra normalized at the 1 b_1 peak of water. **b)** The contributions from the sulfite, bisulfite, and sulfonate are enlarged (grey area) in b). **c)** We used up to three Gaussians to fit this part of the spectrum.

1.4 Concentration profiles

We have investigated the depth distribution of the solvated sulfur ions using two different XPS-based methods. These experiments were performed for solutions at pH = 3.8 and pH = 7.0. The results are shown in Fig. 6. We recorded depth profiles in the traditional manner, by varying the kinetic energy of the photoelectrons between 100 and 1000 eV, which leads to different probing depths due to variation in the inelastic mean free path of the electrons (left panels in Fig. 6). One has to bear in mind, however, that the signal is always integrated from the interface into the bulk, no matter what the overall probing depth is. In addition, we have also recorded photoelectron angular distributions (PADs), which are sensitive to the elastic-scattering mean free path of the photoelectrons. In this method XPS spectra are recorded at different angles (0 to 90 deg) between the electric-field vector of the incident (linearly polarized) X-rays with respect to the electron detection direction. A plot of the intensity of the XPS peaks of the different species as a function of angle allows to fit for the beta parameter for each species. The deeper the species is on average located in the solution,

the smaller the beta parameter. For details on this method we refer to several recent publications.[8–11]

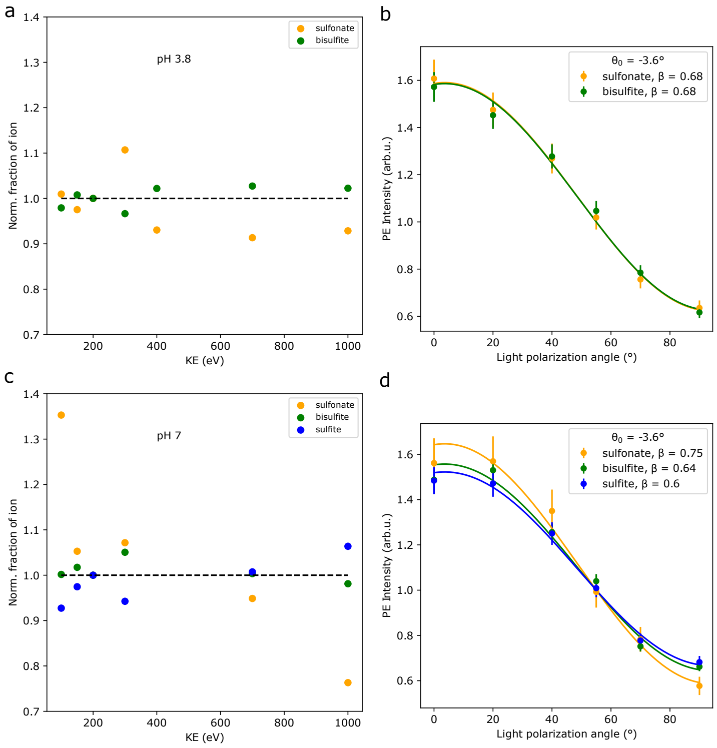

Supplementary Figure 6 Concentration profiles of sulfonate, bisulfite, and sulfite determined by **(a,c)** variation of the kinetic energy, and **(b,d)** by measuring the photoelectron angular distributions, which are a measure of the elastic scattering of photoelectrons and thus of the depth of each species into solution. The upper row shows measurements at pH 3.8, the lower at pH 7.0. Both methods give the same results. In the case of the solution at pH 3.8, the sulfonate and bisulfite species are evenly distributed throughout the solution. At pH 7.0, there is a slight surface enhancement of sulfonate over the bisulfite and sulfite species. The fractions in **(a)** and **(c)** are normalized to the value in the spectra at KE = 200 eV, which are discussed in the main manuscript. The beta parameters in **(b)** and **(d)** are calculated from PAD measurements at a kinetic energy of 100 eV.

The results of the fits of the beta parameters for the different species are shown in the right panels of Fig. 6. Both methods indicate that at pH 3.8, where only sulfonate and bisulfite are present in solution, the two species show a similar depth distribution, with no discernible enrichment of one over the other at the interface. The conclusion can be drawn from the similar ion fraction at the lowest kinetic energies (KEs) in the KE-dependent measurements (Fig. 6a), while the similarity of the PADs (Fig. 6b) points to an overall comparable depth distribution. The situation is different for the case of pH 7.0, where both the KE-dependent measurements (Fig. 6c) as well as the

PADs (Fig. 6d) show a slight enhancement of sulfonate over the sulfite and bisulfite species.

2 Raman spectroscopy

Raman spectra of sulfite at a few pHs were recorded by Eldridge et al. as well as Risberg et al., who also assigned the Raman modes.[12, 13] Here, we focus on the region between 800 and 1300 wavenumbers, which includes features of all species. Example fits at four different pH values are shown in Fig. 7. From the ratios of the symmetric stretch S-O band of sulfite (dark blue), bisulfite (dark green), sulfonate (orange), and sulfur dioxide (purple) we calculated the peak ratios shown in the main manuscript. Furthermore, we checked if the Raman spectra are changing over time, as the XPS spectra cannot be recorded directly after preparation (see Fig. 8). We did not observe significant changes here over time.

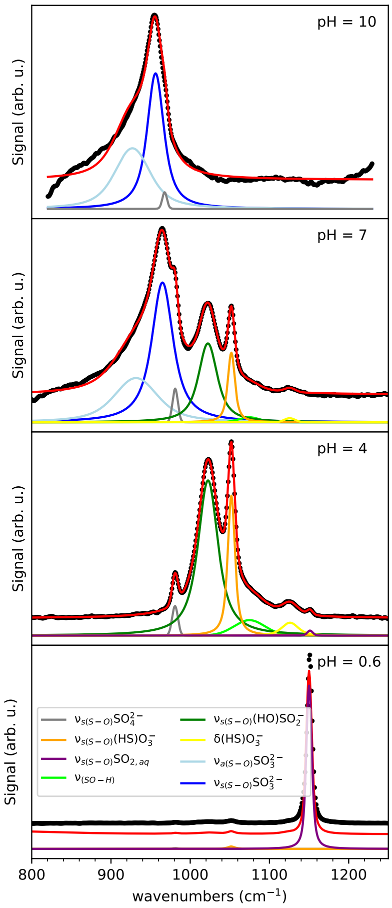

Supplementary Figure 7 Example fits of Raman spectra at four different pHs. The peak assignments are based on the work of Risberg et al.[13].

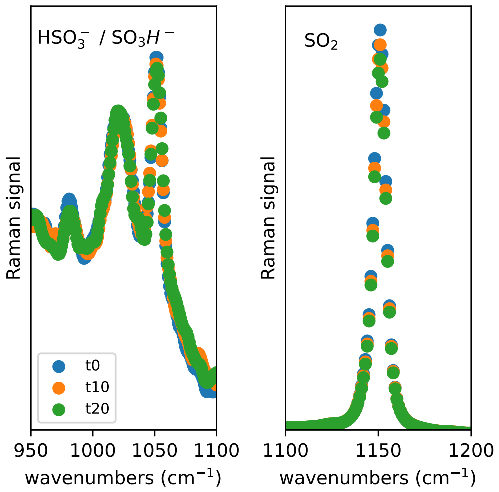

Supplementary Figure 8 Raman spectra of 400 mM sulfite solution at pH=1 at different times (0 min, 10 min, and 20 min after adjusting the pH). There are no significant differences in the spectra.

3 Computational methodology and results

3.1 Classical molecular dynamics

Classical molecular dynamics (MD) simulations were employed to determine the bulk vs. interfacial propensity of sulfurous acid (SO_3H_2), sulfonate (HSO_3^-), bisulfite (SO_3H^-) and sulfite (SO_3^{2-}) ions, as well as SO_2. Classical MD simulations rely on a force field, i.e., a set of parameters and functional forms describing all the inter- and intramolecular interactions in the modelled system. In this work we followed the generalized AMBER force field, GAFF2[14], practice. First, the solute structures were optimized by the MP2/6-31g(d) quantum calculations. Starting from the optimized structures, atomic partial charges were determined by fitting the electrostatic potential obtained at the HF/6-31g* level using the Restrained Electrostatic Potential (RESP) method with a Merz-Singh-Kollman scheme.[15] Quantum calculations were conducted using Gaussian16[16], and the RESP calculations by Antechamber[17]. Other bonded and nonbonded (i.e., van der Waals) interactions were adopted from GAFF2.[14] Similar procedures were used elsewhere for simulations of other aqueous solutions with organic (and not) solutes.[18, 19] TIP3P[20], which is the reference water model for GAFF2, was used to describe the liquid phase. The partial atomic charges of our ionic species (i.e., HSO_3^-, SO_3H^- and SO_3^{2-}) were scaled-down by a factor of 0.75. The "scaling down" of the ionic charges is a convenient way to include electronic polarizability in nonpolarizable MD, allowing polarizable MD simulations at the cheaper computational cost of nonpolarizable ones.[21, 22] The partial charges of the other species in solution (i.e., water, SO_3H_2, and SO_2) were not scaled down.

A water slab was created starting from a bulk liquid water system of initial dimensions of 3 nm, 3 nm, and 4 nm in the x, y, and z directions, respectively, comprising of 574 water molecules, 1 SO_3H_2, 1 HSO_3^-, 1 SO_3H^-, 1 SO_3^{2-}, and 1 SO_2. Na^+ ions were added to neutralize the total system charge. Na^+ force field parameters were adopted from the GAFF2 force field[14] and its ionic charge was also scaled down to +0.75e. This bulk liquid water solution was equilibrated for 1 ns using a constant temperature and pressure (NpT) run at 300 K and 1 bar pressure. Afterwards, the z dimension of the box was enlarged up to 7.2 nm, resulting in a liquid water slab with two water-vapor interfaces. The molar concentrations of the sulfur species in solution correspond, for each species, to 0.1 M. Finally, 400 ns constant volume and temperature (NVT) runs were performed starting from the water slab configuration. For the sake of comparison with our experimental data, we also performed an independent classical MD run solvating one sulfate ion and 2 Na^+ ions in a water slab, following the procedure outlined above.

The coordination number (CN), i.e., the number of contacts between the sulfur (S) atom of SO_2 with the oxygen (O) atoms in the system, was used to discriminate between aqueous $SO_{2(aq.)}$ and gas-phase $SO_{2(g)}$. This metric is defined as:

$$CN = \sum_{i \in O} n(r_{i,S}) \tag{8}$$

with

$$n(r) = \frac{1 - (r/r_c)^p}{1 - (r/r_c)^q} \tag{9}$$

Here, $r_{i,S}$ denotes the distance between each i-O atom in the system and the S atom. r_c is a cutoff distance, which is set to 0.2 nm in this particular case. p and q were set to 10 and 20, respectivily. The n(r) exhibits a value close to 0.9 when the S-O pair distance is below r_c, smoothly converging to zero for distances above the cutoff.

Figure 9 shows the 2D distribution obtained collecting the CN and the distance of the sulfur atom of SO_2 from the Gibbs Diving Surface (GDS, dashed yellow line) over 400 ns classical MD simulation. The funnel shape suggests a CN threshold (orange dashed line) to discriminate between gas phase and aqueous solvated SO_2, a threshold that has been used in Figure 3a to identifies $SO_{2(aq.)}$ and $SO_{2(g)}$. Specifically, a CN$\approx$ 1.84 characterizes gas-phase SO_2, while CN exceeds 1.87 for SO_2 solvated in solution. It is important to stress that in classical and non-reactive MD reaction chemical bonds cannot form or break, contrary to first-principle MD (described below). The GDS was defined as described in Ref. [23].

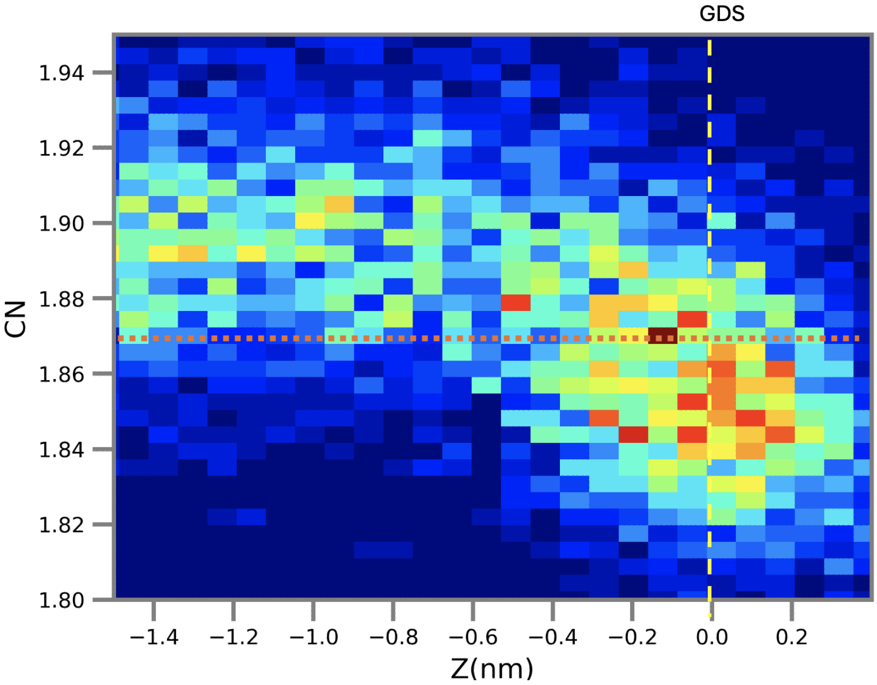

Supplementary Figure 9 2D (not normalized) distribution obtained colleting the CN and the distance, Z, of the S atom of SO_2 from the Gibbs Diving Surface, GDS, (dashed yellow line) over 400 ns classical MD simulation.

All classical MD simulations were performed using GROMACS 2018,[24] and a leapfrog integrator[25] with a time step of 1 fs. Standard 3D periodic boundary conditions were applied. The temperature was controlled at 300 K using the stochastic

velocity rescale thermostat[26] with a coupling time of 0.1 ps. For the NpT equilibration runs, the Parrinello-Rahman barostat[27] was adopted with a time coupling constant of 2 ps and a water compressibility of $4.6 \cdot 10^{-5}$ bar^{-1}. For the nonbonded (real-space Coulomb and van der Waals) interactions we used a cutoff distance of 1.0 nm, while the long-range parts were evaluated using the Particle-Mesh Ewald method[28] with a relative tolerance of 10^{-5}, fourth order cubic interpolation, and a Fourier spacing parameter of 0.12. Finally, the intramolecular bonds in the solutes were constrained using LINCS,[29] while SETTLE[30] was used for constraining the O-H bonds in the water molecules.

3.2 First-principle molecular dynamics

Molecular-dynamics simulations at a first-principles level (FPMD), i.e., with forces driving the dynamics calculated *"on-the-fly"* using density functional theory, were performed to determine *(a)* the stability of the SO_3H_2 dimer, *(b)* sulfurous and sulfonic acid dissociation, and *(c)* sulfonate and bisulfite dehydration at the water-vapor interface. A liquid-water box of 216 water molecules and dimension of $1.5 \times 1.5 \times 3.2$ nm^3 was equilibrated at 1 bar pressure and 300 K using classical MD and the TIP3P water model,[20] adopting the equilibration protocol described above. After equilibration, the z dimension of the simulation box was enlarged to 7.2 nm, resulting in a water slab system with two vapour-liquid water interfaces.

FPMD were carried out using the CP2K molecular dynamics package[31] and the gaussian and plane waves (GPW) method implemented in CP2K. The PBE[32] density functional theory with the Grimme dispersion correction (D3)[33] was adopted. Valence electrons were described by a DZVP basis set, while core electrons were treated by Goedecker–Teter–Hutter pseudopotentials.[34] The cutoff was set to 600 Ry. The temperature was kept at 300 K using a Nose thermostat[35] and a time constant of 50 fs. The time step was 0.5 fs. This (or a very similar) computational protocol has been extensively used in the literature for simulation of bulk water and vapour-liquid water interfaces.[36, 37]

3.3 Interfacial stability of sulfurous acid (SO_3H_2) dimer

In the investigation of the interfacial stability of the sulfurous acid (SO_3H_2) dimer, the dimer geometry was initially optimized in vacuum and afterward placed at one of the two interfaces. The slab system containing the interfacial dimer underwent further optimization through energy minimization, followed by a constant volume and temperature (NVT) FPMD run. The results are displayed in Figure 10, revealing a poorly-stabilized dimer at the aqueous-vapor interface.

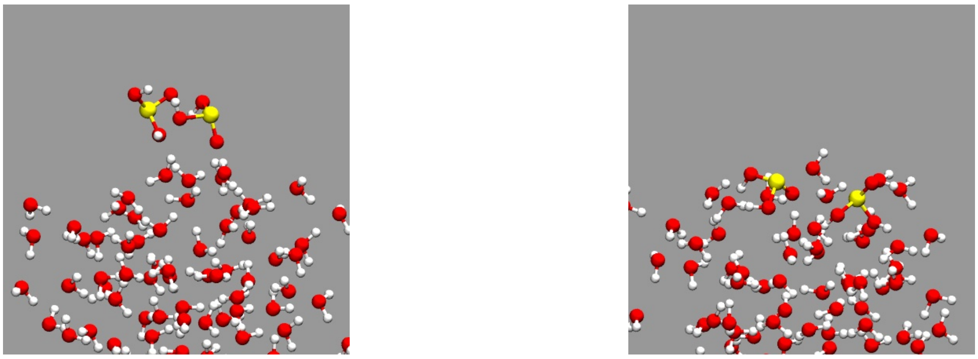

Supplementary Figure 10 Stability of sulfurous acid at the vapor-liquid water interface at 300 K. Left panel: starting optimized configuration. Right panel: after 3.5 ps FPMD, showing the two SO_3H_2 moving apart.

3.4 GW calculations

Experimental XPS data can be compared to theoretically calculated core-electron binding energies (CEBEs) by virtue of Koopmans' theorem,[38],i.e., BEs as the core ionization energies. We here employed the GW method, which is now becoming the gold standard also for core-level predictions[39–41]because it substantially improves the description of the correlation and the exchange interactions between the electrons and the holes created upon photoemission compared to other methods.[42]

Due to the computational cost of the GW method, calculations were performed on molecular clusters carved from the classical and FPMD trajectories around the species under investigation. For each solute, we determined the MD frames in which the compound was at the very interfacial regions, defined here as above the GDS, i.e., toward the gas phase. Thus, we selected at least 50 snapshots, with a minimum time-spacing of 1 ns between them to avoid structural correlations, using a spherical cutoff of 0.45 nm around the solute. On average, each cluster consisted of approximately 50 atoms, depending on the considered molecule. Each cluster geometry extracted from the MD trajectory was then optimized at PBE-D3[32, 33, 43] density functional theory level in vacuum, using a Martyna Tuckerman (MT) solver.[44] After optimization, we computed the binding energies (BEs) on top of the optimized geometry. A similar cluster protocol has been already employed in combination with GW calculations in the literature. [40] It is worth noting that a certain number of MD frames show pairing between different sulfur species (e.g., between sulfonate and bisulfite). These frames represent less than 2% of the total trajectories and were not considered in the calculation of the BEs.

GW calculations were conducted using two different approaches. In the first approach, we employed the eigenvalue self-consistent GW scheme (evGW), starting from the PBE eigenenergies, as described in Ref. [45]. All electron calculations were performed using the Gaussian and Augmented Plane Waves (GAPW) scheme implemented in CP2K, using the def2QZVP basis set and the RI-5Z auxiliary basis [46]. Relativistic effects were accounted for via the zero-order regular approximation

Table 1 Fitting results of photoelectron spectroscopy experiments, BE, and results from GW2X calculations, BE_{theo}. All values are given in eV. The measured binding energy of the SO_2 gas phase peak matches the literature value.[50] In addition to sulfite solutions, we also measured sodium sulfate and methyl-sulfonate solutions (Fig. S1 and S2). The spin-orbit-splitting was in all cases 1.2 eV. Experimental and calculated values are in agreement. The error bar is reported as one standard deviation.

Species	BE	Δ BE	width	BE_{theo}	Δ BE_{theo}
SO_3^{2-}	171.2	0	0.9±0.03	165.5±0.3	0
SO_3H^-	172.0	0.8	0.96±0.02	168.7±0.3	3.2±0.4
HSO_3^-	173.4	2.1	1.15±0.02	170.2±0.4	4.7±0.5
$SO_{2(aq.)}$	174.7	3.5	0.7±0.1	173.0±0.8	7.6±0.9
$SO_{2(g)}$	174.7	3.5	0.23±0.08	174.2±0.8	8.7±0.8
SO_4^{2-}	173.2	2.0	0.93±0.05		
H_2SO_3				172.3±1.4	6.8±0.6
HSO_3H				173.0±0.4	7.5±0.5
Me-SO_3^-	173.5	2.3	0.93±0.02		

(ZORA) to the Dirac equation [47]. In the second approach, we utilized the GW2X method as implemented[48] in CP2K, starting from the PBE0[49] eigenenergies and employing GAPW with Ahlrichs-def2-TZVP basis sets.[46]. For both GW approaches, the energy cutoff for the plane waves was set to 600 Ry, and Goedecker–Teter–Hutter pseudopotentials[34] were employed to treat the core electrons of non-sulfur atoms in the systems (i.e., O and H atoms).

While the evGW method provides more reliable core-level predictions in terms of absolute binding energy, the GW2X method better describes the Spin-Orbit Coupling (SOC) effect, which is relevant for the $2p_{1/2}$ and $2p_{3/2}$ line splitting. However, the latter may lead to a systematic shift in the absolute binding energies compared to experimental values.[39] Nevertheless, within statistical uncertainty, both methods predict similar relative BE differences and the same BE trend among the different species, as shown by comparing Table S1 and Table 1 in the main manuscript.

3.5 Acid dissociation mechanism at the liquid-vapor interface

We investigated the acid dissocition of sulfurous acid and sulfonic acid to bisulfite and sulfonate, respectively, employing FPMD coupled with on-the-fly probability enhanced sampling metadynamics[51] (hereafter referred to as OPES-MTD) and the weighted histogram analysis method (WHAM)[52]. Molecular dynamics simulations were performed using CP2K[31] with the PLUMED plug-in[53]. Due to the cost of simulating multi reaction mechanisms at the air-liquid water interface by FPMD, here we adopted a cluster approximation of the interfacial environment, i.e., we used snapshot carved from the classical MD trajectory of the solute at the air-liquid water interface as starting configurations of our FPMD. The rational of this choice is that in the timeframe of our FPMD ($\approx$ 10 ps), clusters of sufficient size do not have enough time to rearrange in a configuration that significantly differ from that at the interface.The starting configurations were extracted from the classical MD trajectory with sulfurous (or sulfonic) acid solvated at the air-liquid water interface using a spherical cutoff radius of

0.9 nm centered on the S atom. The cutoff radius was larger than the radius of the second solvation shell between the oxygen atoms of the acids and the water oxygens (see Figure 3b of main manuscript). Moreover, Baer et al.,[36] has revised the properties of the vapor-liquid water interface by FPMD reporting a thickness of the interface environment of approximately 0.5 nm at 300 K, smaller than the cutoff here adopted (i.e., 0.9 nm). Indeed, looking at the radial distribution function (RDF) between the oxygen of the sulfurous species and the water oxygen in Figure 3b, we observe a saturation of the RDF at ≈0.6 nm from the interfacial S atom, as a sign of a bulk like behavior. Thus, we are confident that our carved clusters are reliable initial configurations comprising of a single sulfurous (or sulfonic) acid solvated on an interfacial environment with at least two solvation shells.

From these initial configurations, two short NVT simulations (one for sulfurous acid and one for sulfonic acid) of 5 ps each were performed centering the structures in a cubic box with dimensions of 3.2 nm, and conducted without periodic boundary conditions using a Martyna-Tuckerman Poisson solver[54]. Throughout the 5 ps NVT runs, the acids remained undissociated. The final frames of the two NVT runs were utilized to start the OPES-MTD. The system size of these starting configurations enabled sufficiently sampled FPMD simulations while still being representative of the solvation environments of the two acids at the liquid-vapor interface.

The acid dissociation was investigated employing two reaction coordinates, also referred to as collective variables (CVs), which were inspired from previous work[55]. The two CVs are:

- n_a, i.e., the number of hydrogen atoms (H) chemically bonded to the oxygen atoms of the acid (Os),
- d_h, i.e., the distance between the hydronium ion and the closest Os of the acid (i.e., sulfurous or sulfonic acid)

n_a was defined using a switching function of the atomic distances between the H and Os atoms in the system:

$$n_a = \sum_{i \in Os, j \in H} n(r_{i,j}) \tag{10}$$

where n is defined in Eq. 9, and $r_{i,j}$ is the distance between each pair of the Os and H atoms.

Here, the coordination number defined in Eq. 9 and Eq. 10 has been used to identify H atoms chemically bonded to the oxygen atoms, O_s, of the acids. For each H atom chemically bonded with one O, n assumes a value of approximately 0.9. n is approximately 0.2 when a H atom is within the first solvation shell of one O, while n values smaller than 0.2 correspond to H atom beyond the first solvation shell (i.e, H_3O^+ solvent-separated from the conjugated ion). In this way, n_a ≈1.8 for sulfurous acid and ≈0.9 for bisulfite. Similarly, n_a is approximately 0.9 for sulfonic acid and ranges from 0.1 to 0.2 for sulfonate ions.

Since in water the excess proton can diffuse by Grotthuss mechanism in water, we identify the hydronium oxygen as the water oxygen, Ow, coordinated with three hydrogens. Then, the distance between the hydronium oxygen and the closest Os of the acid was defined as

Table 2 CV settings.

	Sulfurous Acid/Bisulfite	Sulfonic Acid/Sulfonate
(p,q)	(10,20)	(10,20)
r_c	0.127 nm	0.127 nm
λ	20	20
n_c	1.55	0.56

$$d_h = f(n_a)\frac{\sum_{i\in Ow} r_i e^{\lambda n_i}}{\sum_{i\in Ow} e^{\lambda n_i}} \tag{11}$$

where r_i is the distance of O_w from the closest O_s of the acid, while n_i is the number of H atoms coordinated with O_w. The distance d_h defined above is a derivable function of the atomic coordinates, which makes it suitable to be used in combination with OPES-MTD. For sufficiently large λ, d_h, the fraction term on the right side of Eq. 11 converges distance between the three-hydrogen coordinated O_w (i.e., the hydronium ion) and the closest Os. The pre-factor $f(n_a) = (1-(n_a/n_c)^p)/(1-(n_a/n_c)^q)$ is a switching function that sets d_h to zero when the acid is protonated.

d_h and n_a are two derivable functions of the atomic coordinates. While n_a describes the protonation/deprotonation of the acid, d_h is necessary to distinguish between contact ion pairing (CIP) configurations (i.e., when the hydronium ion is still in contact with the deprotonated base) and dissociated products (i.e., when the base and the hydronium ion are solvent-separated). Table S2 contains the parameters utilized for d_h and n_a. These CVs and parameters were inspired by previous literature reports on acetic acid dissociation[55] and tuned for our 5 ps NVT unbiased MD runs.

OPES-MTD simulations were conducted by sampling the bias landscape in the d_h and n_a space, using a kernel frequency deposition of 50 steps (i.e., 25 fs). Figure 11 and 12 illustrate the 2D bias landscape for deprotonation of sulfurous and sulfonic acid obtained from 7 ps MTD runs. In 11, three distinct minima can be identified: one corresponding to sulfurous acid at $(d_h, n_a) = (0.07, 1.86)$, another to the contact ion pairing (CIP) between the deprotonated acid and the hydronium ion $(d_h, n_a) = (0.27, 1.14)$, and a third one for the solvent separated ions at $(d_h, n_a) = (0.73, 1.11)$. Similarly, Figure 12 displays a minimum for sulfonic acid at $(d_h, n_a) = (0.01, 0.86)$, along with a larger minimum encompassing both CIP and solvent-separated products. It is important to stress that, due to inherent sampling limitations within current FPMDs, Figure 11 and 12 should be interpreted as a 2D bias landscape, i.e., regions that were most frequently visited and, thus, bias potential deposited, rather than a fully sampled 2D free energy map. Nonetheless, these 2D bias landscapes are highly valuable for extracting information about potential new pathways and intermediates.

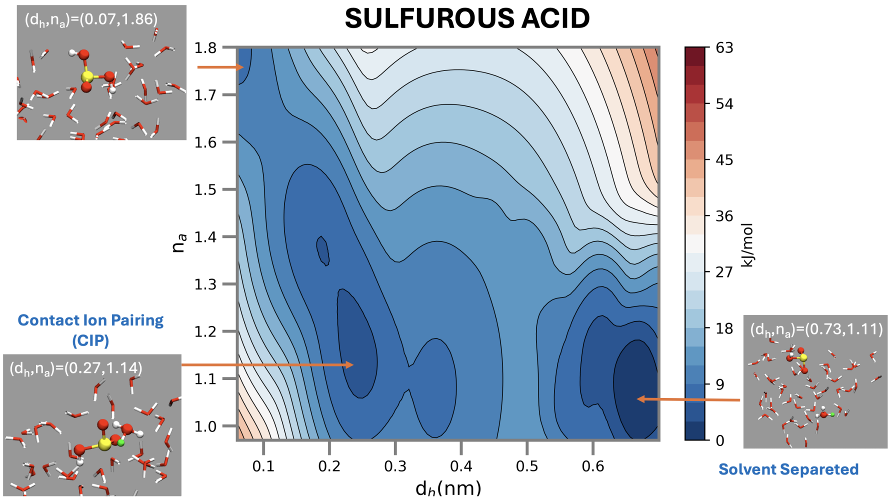

Supplementary Figure 11 2D bias landscape in the d_h and n_a space for sulfurous acid dissociation obtained from the MTD simulation.

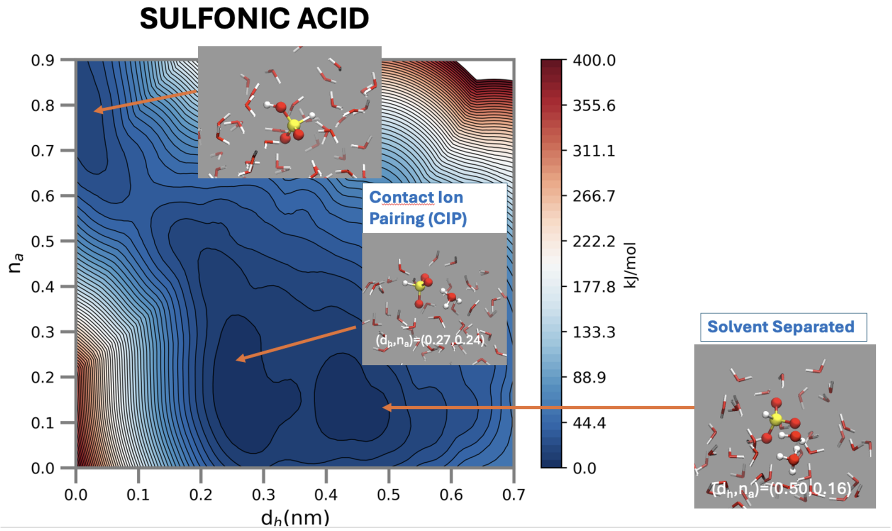

Supplementary Figure 12 2D bias landscape in the d_h and n_a space for sulfonic acid dissociation obtained from the MTD simulation.

The 2D maps in Figure 11 and 12 show that, specifically in the cases of sulfurous and sulfonic acid, the variable d_h alone effectively characterizes the entire process from the undissociated acid to the contact ion pairing (CIP) and onward to the solvent-separated configuration. Supporting this observation, Figure 13 illustrates the number of water molecules (q_{SOL}) coordinating the hydronium ion as a function of the distance d_h. In solution, a water molecule typically forms four hydrogen bonds, whereas

a hydronium ion forms only three. For both acids, $q_{SOL} \approx 3$ for $d_h > 0.5nm$, indicating a well-solvated hydronium ion at a sufficient distance from the dissociated base. Consequently, the 2D maps in Figure 11 and 12 were refined using WHAM[52] to yield a more thoroughly sampled 1D profile along the d_h coordinate.

We refined the 2D map reconstruction by WHAM to generate a 1D free energy profile along the d_h coordinate. For both acids, we selected six configurations from the OPES-MTD, with d_h intervals of 0.1 nm ranging from d_h=0.1 nm to d_h=0.6 nm. For each configuration, we conducted 5 ps FPMD applying a harmonic restraint potential of k=1500 kJ/mol centered on the initial d_h value. Additionally, to ensure better overlap of the umbrella histograms in the region of steepest free energy (e.g., in proximity of the transition state), we performed two additional windows at d_h=1.5 nm and d_h=5.5 nm using K = 6000 kJ/mol. Error bars on the 1D free energy profiles were calculated using block averaging[56] over two blocks and are shown in Figure 3 as shadow regions. Specifically, we used the first and last halves of each umbrella trajectory after discarding the initial 1 ps of each trajectory for equilibration purposes. The above-outlined procedure follows similar protocols in literature for the calculation of free energy profiles for chemical reactions by FPMD.[37]

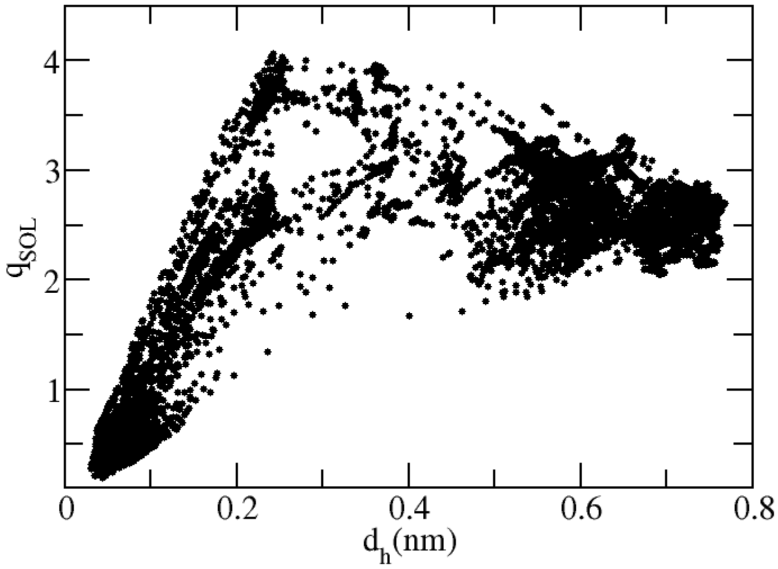

Supplementary Figure 13 The number of water molecules (qsol) coordinating the hydronium ion as a function of the distance, dh, between the hydronium ion and the closest oxygen atom, Os, of the acid molecule (i.e., sulfurous or sulfonic acid). The q_{sol} value was calculated based on the distance between the hydronium oxygen and the water oxygen, Ow, employing Eq. 9 in the SI, with a cutoff threshold of 0.29 nm.

The 1D free energy profiles for the acid dissociation are shown and commented on in Figure 3 of the main manuscript. These profiles reveal nearly barrierless deprotonation for both sulfonic and sulfurous acids, where the deprotonated bases are thermodynamically favored (i.e., at lower energy) compared to the undissociated acids. However, for sulfonic acid (depicted by the yellow line), the global minimum corresponds to contact ion pair formation between sulfonate and the hydronium ion.

3.6 Dehydration mechanism at the liquid-vapor interface

We investigated the dehydration process of sulfonate and bisulfite ions at the liquid-vapor interface, employing a protocol closely resembling that used for acid dissociation, as described above. As suggested by the acid dissociation profiles in Figure 3b, both sulfurous and sulfonic acids rapidly undergo deprotonation, leading to the formation of sulfonate and bisulfite ions at the liquid-vapor interface. Thus, we investigated the hydration process starting from the solvated ions, using the coordination number (CN) as reaction coordinate, which quantifies the number of contacts between the sulfur (S) and oxygen (O) atoms in the system, as described by Eq. 8. The cutoff distance was set to r_c=0.19 nm and (p,q)=(8,16). CN is a derivable function that takes a value of $\approx$ 0.9 for each S-O covalently bonded. Thus, sulfonate and bisulfite correspond to a CN $\approx$2.7 (i.e., 3 S-O bonds). For SO_2 in solution CN is around 2.0. This slightly higher value than 1.8 (i.e., 2 S-O bonds) is caused bythe contribution of water oxygens in the solvation shell of SO_2.

We performed FPMD coupled with metadynamics[57], MTD, to obtain a preliminary bias landscape of the process and identify key intermediate structures. The starting configuration for the MTD runs was extracted from the classical MD trajectory with sulfonate (or bisulfite) using a spherical cutoff radius of 0.6 nm centered on the S atom, resulting in a water cluster of 12 water molecules with sulfonate (or bisulfite) solvated on top of it. While a cluster approach is an unavoidable approximation of the interfacial environment due to the computational cost of FPMD, a cluster size of 0.6 nm is large enough to catch the solute in an environment resembling those of the air-liquid water interface. Also keeping in mind that in the timeframe of our FPMD ($\approx$ 10 ps), clusters of sufficient size do not have enough time to rearrange in a configuration that significantly differ from that at the interface from where they were carved. (see also mentioned in section 3.5) This starting configuration was then centered in a cubic box of 2 nm side lengths, and simulations were performed without periodic boundary conditions using a Martyna-Tuckerman Poisson solver.[54] During the MTD runs, a bias Gaussian potential of σ-width 0.2 nm and height of 4.148 kJ/mol was deposited every 100 steps (i.e., every 50 fs).

The 1D free energy profiles along the CN coordinate in Figure 3c were constructed by WHAM[52] starting from MTD trajectory frames at different CN-values. We extracted 10 configurations with CN ranging from CN=1.9 to CN=2.8. For each frame, we conducted 10 ps of FPMD, constraining CN to its initial value by applying a harmonic restraint potential with a force constant of k=1500 kJ/mol. In the specific case of sulfonate and for the sake of obtaining better overlapping histograms, we added three additional umbrella windows at CN=2.05, 2 .2, 2.3 using a k=3000

kJ/mol. Finally, the restrained trajectories were used to generate the 1D free energy profile in Figure 3c by WHAM.[52]

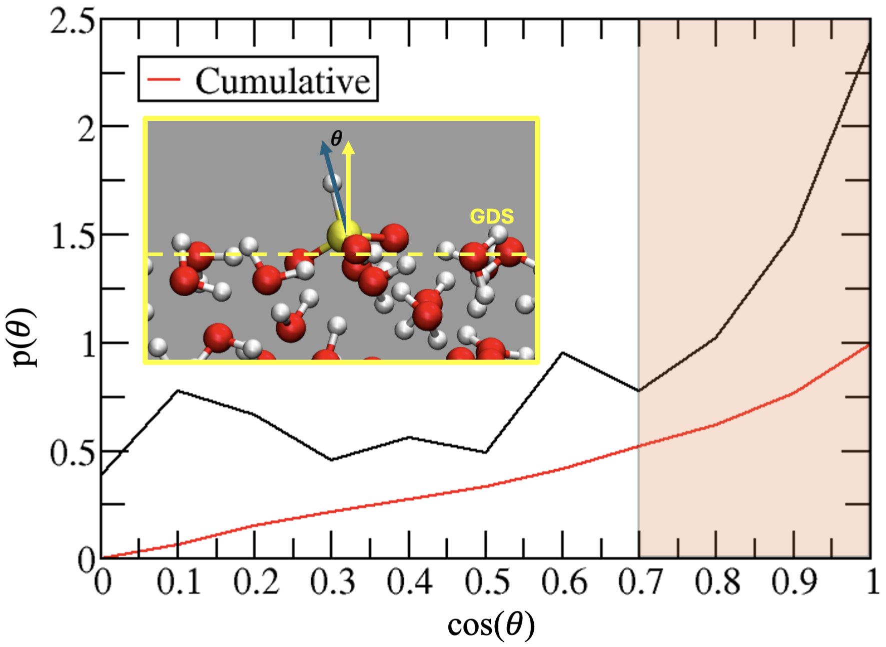

Supplementary Figure 14 Probability distribution for the $cos(\theta)$ collected over 400 ns classical MD trajectory. Here, θ represents the angle between the S-H intramolecular bond and the normal to the Gibbs Dividing Surface, GDS.The cumulative distribution is depicted in red. The shaded red region emphasizes that for more than 50 % of the trajectory frames, $cos(\theta) \geq 0.7$, which implies $0° < \theta < 45°$.

References

[1] Henry, W. Iii. experiments on the quantity of gases absorbed by water, at different temperatures, and under different pressures. Phil. Trans. **93**, 29–274 (1803). URL https://doi.org/10.1098/rstl.1803.0004.

[2] Lide, D. R. Handbook of Chemistry and Physics, 84th Edition (Taylor and Francis, 2004).

[3] Sander, R. Herny's law constants. URL https://henrys-law.org/henry/casrn/7446-09-5.

[4] Buttersack, T. et al. Imaging temperature and thickness of thin planar liquid water jets in vacuum. Struct. Dyn. **10**, 034901 (2023). URL https://10.1063/4.0000188.

[5] Scholze, F. et al. High accuracy detector calibration for euv metrology at ptb. Prodceedings SPIE **4688**, 680–689 (2002). URL https://doi.org/10.1117/12.472342.

[6] Yeh, J. & Lindau, I. Atomic subshell photoionization cross sections and asymmetry parameters: $1 \leqslant z \leqslant 103$. Atomic Data and Nuclear Data Tables **32**, 1–155 (1985). URL https://www.sciencedirect.com/science/article/pii/0092640X85900166.

[7] Winter, B., Aziz, E. F., Hergenhahn, U., Faubel, M. & Hertel, I. V. Iii. experiments on the quantity of gases absorbed by water, at different temperatures, and under different pressures. J. Phys. Chem. **126**, 124504 (2005). URL https://doi.org/10.1063/1.2710792.

[8] Thürmer, S. et al. Photoelectron angular distributions from liquid water: Effects of electron scattering. Phys. Rev. Lett. **111**, 173005 (2013). URL https://link.aps.org/doi/10.1103/PhysRevLett.111.173005.

[9] Dupuy, R. et al. Angstrom-depth resolution with chemical specificity at the liquid-vapor interface. Phys. Rev. Lett. **130**, 156901 (2023).

[10] Dupuy, R. et al. Core-level photoelectron angular distributions at the liquid–vapor interface. Acc. Chem. Res. **3**, 215–223 (2023). URL https://pubs.acs.org/doi/10.1021/acs.accounts.2c00678.

[11] Dupuy, R. et al. Core level photoelectron spectroscopy of heterogeneous reactions at liquid–vapor interfaces: Current status, challenges, and prospects. The Journal of Chemical Physics **154**, 060901 (2021).

[12] Eldridge, D. L., Mysen, B. O. & Cody, G. D. Experimental estimation of the bisulfite isomer quotient as a function of temperature: Implications for sulfur isotope fractionations in aqueous sulfite solutions. Geochimica et Cosmochimica Acta **220**, 309–328 (2018).

[13] Risberg, E. D. et al. Sulfur x-ray absorption and vibrational spectroscopic study of sulfur dioxide, sulfite, and sulfonate solutions and of the substituted sulfonate ions $x_3cso_3^-$ (x = h, cl, f). Inorganic Chemistry **46**, 8332–8348 (2007).

[14] Jämbeck, J. P. M. & Lyubartsev, A. P. Update to the general amber force field for small solutes with an emphasis on free energies of hydration. J.Phys. Chem. B **118**, 3793–3804 (2014).

[15] Bayly, C. I., Cieplak, P., Cornell, W. & Kollman, P. A. A well-behaved electrostatic potential based method using charge restraints for deriving atomic charges: the resp model. J. Phys. Chem. **97**, 10269–10280 (1993).

[16] Frisch, M. J. et al. Gaussian˜16 Revision C.01 (2016). Gaussian Inc. Wallingford CT.

[17] Wang, J., Wang, W., Kollman, P. A. & Case, D. A. Automatic atom type and bond type perception in molecular mechanical calculations. Journal of Molecular Graphics and Modelling **25**, 247–260 (2006). URL https://www.sciencedirect.com/science/article/pii/S1093326305001737.

[18] Gladich, I., Habartová, A. & Roeselová, M. Adsorption, mobility, and self-association of naphthalene and 1-methylnaphthalene at the water–vapor interface. J. Phys. Chem. A **118**, 1052–1066 (2014).

[19] Vácha, R., Slavíček, P., Mucha, M., Finlayson-Pitts, B. J. & Jungwirth, P. Adsorption of atmospherically relevant gases at the air/water interface: Free energy profiles of aqueous solvation of n_2, o_2, o_3, oh, h_2o, ho_2, and h_2o_2. J. Phys. Chem. A **108**, 11573–11579 (2004).

[20] Jorgensen, W. L., Chandrasekhar, J., Madura, J. D., Impey, R. W. & Klein, M. L. Comparison of simple potential functions for simulating liquid water. J. Chem. Phys. **79**, 926–935 (1983).

[21] Leontyev, I. & Stuchebrukhov, A. Accounting for electronic polarization in non-polarizable force fields. Phys. Chem. Chem. Phys. **13**, 2613–2626 (2011).

[22] Vazdar, M., Pluhařová, E., Mason, P. E., Vácha, R. & Jungwirth, P. Ions at hydrophobic aqueous interfaces: Molecular dynamics with effective polarization. J. Phys. Chem. Lett. **3**, 2087–2091 (2012).

[23] Yang, H., Gladich, I., Boucly, A., Artiglia, L. & Ammann, M. Orcinol and resorcinol induce local ordering of water molecules near the liquid–vapor interface. Environ. Sci.: Atmos. **2**, 1277–1291 (2022).

[24] Abraham, M. J. et al. Gromacs: High performance molecular simulations through multi-level parallelism from laptops to supercomputers. SoftwareX **1-2**, 19–25 (2015). URL https://www.sciencedirect.com/science/article/pii/S2352711015000059.

[25] Hockney, R., Goel, S. & Eastwood, J. Quiet high-resolution computer models of a plasma. J. Comp. Phys. **14**, 148–158 (1974). URL https://www.sciencedirect.com/science/article/pii/0021999174900102.

[26] Bussi, G., Donadio, D. & Parrinello, M. Canonical sampling through velocity rescaling. J. Chem. Phys. **126**, 014101 (2007).

[27] Parrinello, M. & Rahman, A. Polymorphic transitions in single crystals: A new molecular dynamics method. Journal of Applied Physics **52**, 7182–7190 (1981).

[28] Essmann, U. et al. A smooth particle mesh Ewald method. J. Chem. Phys. **103**, 8577–8593 (1995).

[29] Hess, B., Bekker, H., Berendsen, H. J. C. & Fraaije, J. G. E. M. Lincs: A linear constraint solver for molecular simulations. J. Comp. Chem. **18**, 1463–1472 (1997).

[30] Miyamoto, S. & Kollman, P. A. Settle: An analytical version of the shake and rattle algorithm for rigid water models. J. Comp. Chem **13**, 952–962 (1992).

[31] Kühne, T. D. et al. CP2K: An electronic structure and molecular dynamics software package - Quickstep: Efficient and accurate electronic structure calculations. J. Chem. Phys. **152**, 194103 (2020). URL https://doi.org/10.1063/5.0007045.

[32] Perdew, J. P., Burke, K. & Ernzerhof, M. Generalized gradient approximation made simple. Phys. Rev. Lett. **77**, 3865–3868 (1996). URL https://link.aps.org/doi/10.1103/PhysRevLett.77.3865.

[33] Grimme, S., Antony, J., Ehrlich, S. & Krieg, H. A consistent and accurate ab initio parametrization of density functional dispersion correction (DFT-D) for the 94 elements H-Pu. J. Chem. Phys. **132**, 154104 (2010). URL https://doi.org/10.1063/1.3382344.

[34] Goedecker, S., Teter, M. & Hutter, J. Separable dual-space gaussian pseudopotentials. Phys. Rev. B **54**, 1703–1710 (1996). URL https://link.aps.org/doi/10.1103/PhysRevB.54.1703.

[35] Nosé, S. A molecular dynamics method for simulations in the canonical ensemble. Molecular Physics **52**, 255–268 (1984).

[36] Baer, M. D. et al. Re-examining the properties of the aqueous vapor–liquid interface using dispersion corrected density functional theory. J. Chem. Phys. **135**, 124712 (2011).

[37] Sinopoli, A., Abotaleb, A., Pietrucci, F. & Gladich, I. Stability of a monoethanolamine-co2 zwitterion at the vapor/liquid water interface: Implications for low partial pressure carbon capture technologies. J. Phys. Chem. B **125**, 4890–4897 (2021).

[38] Tardio, S. & Cumpson, P. Practical estimation of xps binding energies using widely available quantum chemistry software. Surf. Inter. Anal. **50**, 5–12 (2018).

[39] Bussy, A. & Hutter, J. Efficient and low-scaling linear-response time-dependent density functional theory implementation for core-level spectroscopy of large and periodic systems. Phys. Chem. Chem. Phys. **23**, 4736–4746 (2021). URL http://dx.doi.org/10.1039/D0CP06164F.

[40] Golze, D. et al. Accurate computational prediction of core-electron binding energies in carbon-based materials: A machine-learning model combining density-functional theory and gw. Chemistry of Materials **34**, 6240–6254 (2022).

[41] Jin, J. L. Y., Rinke, P., Yang, W. & Golze, D. Benchmark of gw methods for core-level binding energies. J. Chem. Theory Comput. **18**, 7570–7585 (2022).

[42] Golze, D., Keller, L. & Rinke, P. Accurate absolute and relative core-level binding energies from gw. J. Phys. Chem. Lett. **11**, 1840–1847 (2020).

[43] Perdew, J. P. et al. Restoring the density-gradient expansion for exchange in solids and surfaces. Phys. Rev. Lett. **100**, 136406 (2008). URL https://link.aps.org/doi/10.1103/PhysRevLett.100.136406.

[44] Martyna, G. J. & Tuckerman, M. E. A reciprocal space based method for treating long range interactions in ab initio and force-field-based calculations in clusters. J. Chem. Phys. **110**, 2810–2821 (1999).

[45] Wilhelm, J., Golze, D., Talirz, L., Hutter, J. & Pignedoli, C. A. Toward gw calculations on thousands of atoms. J. Phys. Chem. Lett. **9**, 306–312 (2018).

[46] Pritchard, B. P., Altarawy, D., Didier, B., Gibson, T. D. & Windus, T. L. New basis set exchange: An open, up-to-date resource for the molecular sciences community. Journal of Chemical Information and Modeling **59**, 4814–4820 (2019).

[47] van Lenthe, E., Baerends, E. J. & Snijders, J. G. Relativistic total energy using regular approximations. J. Chem. Phys. **101**, 9783–9792 (1994). URL https://doi.org/10.1063/1.467943.

[48] Bussy, A. & Hutter, J. First-principles correction scheme for linear-response time-dependent density functional theory calculations of core electronic states. The Journal of Chemical Physics **155**, 034108 (2021). URL https://doi.org/10.1063/5.0058124.

[49] Adamo, C. & Barone, V. Toward reliable density functional methods without adjustable parameters: The PBE0 model. The Journal of Chemical Physics **110**, 6158–6170 (1999).

[50] Siggel, M. R. F., Field, C., Sæthre, L. J., Bo/rve, K. J. & Thomas, T. D. High resolution photoelectron spectroscopy of sulfur 2p electrons in h_2s, SO_2, CS_2, and OCS. J. Chem. Phys. **105**, 9035–9039 (1996).

[51] Invernizzi, M. & Parrinello, M. Rethinking metadynamics: From bias potentials to probability distributions. The Journal of Physical Chemistry Letters **11**, 2731–2736 (2020).

[52] Kumar, S., Rosenberg, J. M., Bouzida, D., Swendsen, R. H. & Kollman, P. A. Multidimensional free-energy calculations using the weighted histogram analysis method. Journal of Computational Chemistry **16**, 1339–1350 (1995).

[53] Tribello, G. A., Bonomi, M., Branduardi, D., Camilloni, C. & Bussi, G. Plumed 2: New feathers for an old bird. Computer Physics Communications **185**, 604–613 (2014).

[54] Martyna, G. J. & Tuckerman, M. E. A reciprocal space based method for treating long range interactions in ab initio and force-field-based calculations in clusters. The Journal of Chemical Physics **110**, 2810–2821 (1999).

[55] Park, J. M., Laio, A., Iannuzzi, M. & Parrinello, M. Dissociation mechanism of acetic acid in water. Journal of the American Chemical Society **128**, 11318–11319 (2006).

[56] Bussi, G. & Tribello, G. A. Analyzing and Biasing Simulations with PLUMED, 529–578 (Springer New York, New York, NY, 2019). URL https://doi.org/10.1007/978-1-4939-9608-7_21.

[57] Laio, A. & Parrinello, M. Escaping free-energy minima. Proceedings of the National Academy of Sciences **99**, 12562–12566 (2002).
